# Induction of release and up-regulated gene expression of interleukin (IL)-8 in A549 cells by serine proteinases

**DOI:** 10.1186/1471-2121-7-22

**Published:** 2006-05-15

**Authors:** Haiyan Wang, Yanshan Zheng, Shaoheng He

**Affiliations:** 1 Allergy and Inflammation Research Institute, the Key Immunopharmacology Laboratory of Guangdong Province, Shantou University Medical College, Shantou 515031, China

## Abstract

**Background:**

Hypersecretion of cytokines and serine proteinases has been observed in asthma. Since protease-activated receptors (PARs) are receptors of several serine proteinases and airway epithelial cells are a major source of cytokines, the influence of serine proteinases and PARs on interleukin (IL)-8 secretion and gene expression in cultured A549 cells was examined.

**Results:**

A549 cells express all four PARs at both protein and mRNA levels as assessed by flow cytometry, immunofluorescence microscopy and reverse transcription polymerase chain reaction (PCR). Thrombin, tryptase, elastase and trypsin induce a up to 8, 4.3, 4.4 and 5.1 fold increase in IL-8 release from A549 cells, respectively following 16 h incubation period. The thrombin, elastase and trypsin induced secretion of IL-8 can be abolished by their specific inhibitors. Agonist peptides of PAR-1, PAR-2 and PAR-4 stimulate up to 15.6, 6.6 and 3.5 fold increase in IL-8 secretion, respectively. Real time PCR shows that IL-8 mRNA is up-regulated by the serine proteinases tested and by agonist peptides of PAR-1 and PAR-2.

**Conclusion:**

The proteinases, possibly through activation of PARs can stimulate IL-8 release from A549 cells, suggesting that they are likely to contribute to IL-8 related airway inflammatory disorders in man.

## Background

Respiratory epithelium acts as the first tissue to meet inhaled pathogens and is capable of releasing inflammatory mediators and cytokines in response. Respiratory epithelial cells can synthesize and secrete a variety of proinflammatory cytokines such as IL-8, IL-1, IL-6, granulocyte-macrophage colony stimulating factor (GM-CSF) [1] and RANTES [2] which regulate cell behavior including growth, secretion, migration in physiological and pathological conditions.

The importance of serine proteinases in the development of airway diseases has been emphasized in recent years. Of particular importance is that the potential role of tryptase [3] thrombin [4] and elastase [5] in the development of asthma, in which these serine proteinases were not only been over-secreted [4,6,7], but also found to play a role in induction of cytokine hypersecretion in airways [8,9]. However, the potential mechanism, through which these serine proteinases carry out their actions in respiratory tract, remains unclear. Since increased level of IL-8 in the airways reported to be closely correlated to asthma [10], we investigated the effect of tryptase, thrombin, trypsin, and elastase on IL-8 secretion and gene expression in A549 cells, a type II alveolar epithelial cell line from human adenocarcinoma, in the present study.

In recent years, PARs have been identified as receptors for serine proteinases. Among them, PAR-1 is a receptor of thrombin and trypsin [11]; PAR-2 is a receptor of trypsin, tryptase [12] and elastase [9]; PAR-3 [13] and PAR-4 [14] are receptors of thrombin. Activation of PARs could profoundly alter secretion ability of numerous cell types such as histamine release from human mast cells [15], IL-6 release from airway epithelial cells [8], IL-1 release from fibroblast [16], and IL-8 release from human oral epithelial cells [17]. We therefore investigated the effect of the agonists of all four types of PARs on IL-8 release from A549 cells in the current study. Since expression of PARs on A549 cells is crucial for the understanding of actions of the serine proteinases tested, we also investigated the expression PAR-1, PAR-2, PAR-3 and PAR-4 on A549 cells with immunocytochemical techniques and reverse transcription polymerase chain reaction (RT-PCR) in the present study.

## Results

### Induction of IL-8 release by serine proteinases

**Figure 1:**
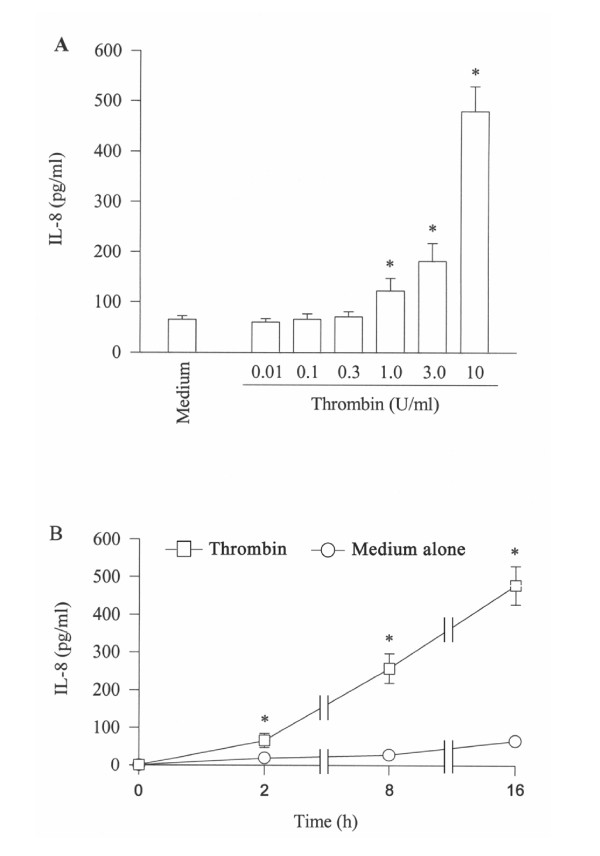
Effect of thrombin on the release of IL-8 from A549 cells. Cells were incubated (**A**) with various concentrations of thrombin at 37°C for 16 h, or (**B**) with 10 U/ml of thrombin for 2 h, 8 h and 16 h. Values shown are mean ± SE for 5 separate experiments. *P < 0.05 compared with the response to medium alone control.

Thrombin at concentrations of 1–10 U/ml provokes a concentration dependent release of IL-8 from A549 cells following 16 h incubation period. Approximately 8 fold increase in IL-8 release is observed at 16 h following incubation with 10 U/ml thrombin (Figure 1A). The time course study shows that increased release of IL-8 induced by thrombin begins within 2 h, and lasts at least to 16 h (Figure 1B).

**Figure 2:**
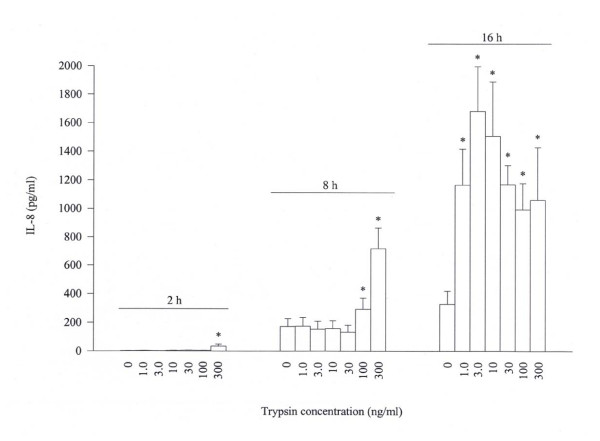
Effect of trypsin on the release of IL-8 from A549 cells. Cells were incubated with various concentrations of trypsin at 37°C for 2 h, 8 h or 16 h. Values shown are mean ± SE for 5 separate experiments. *P < 0.05 compared with the response to medium alone control.

At the concentrations from 1 to 300 ng/ml, trypsin is able to stimulate a 'bell shape' release of IL-8 from A549 cells following 16 h incubation period. The maximum release of 5.1 fold is observed when 3 ng/ml of trypsin was added to A549 cells. At the time of 8 h, however, a dose dependent release of IL-8 from A549 cells is achieved with 100 and 300 ng/ml trypsin. Small but nevertheless significant release of IL-8 is also observed with 300 ng/ml trypsin following 2 h incubation (Figure 2). Also in Figure 2, it is clearly observed that the basal accumulated secretion of IL-8 from A549 cells is time dependent with 2.7 ± 0.7, 173 ± 54 and 329 ± 91 pg/ml being secreted following 2, 8 and 16 h incubation periods, respectively. Trypsin at concentration of 300 ng/ml fails to stimulate IL-10, IL-16, IL-17 and IL-18 secretion from A549 cells following 8 h incubation period (data not shown).

**Figure 3:**
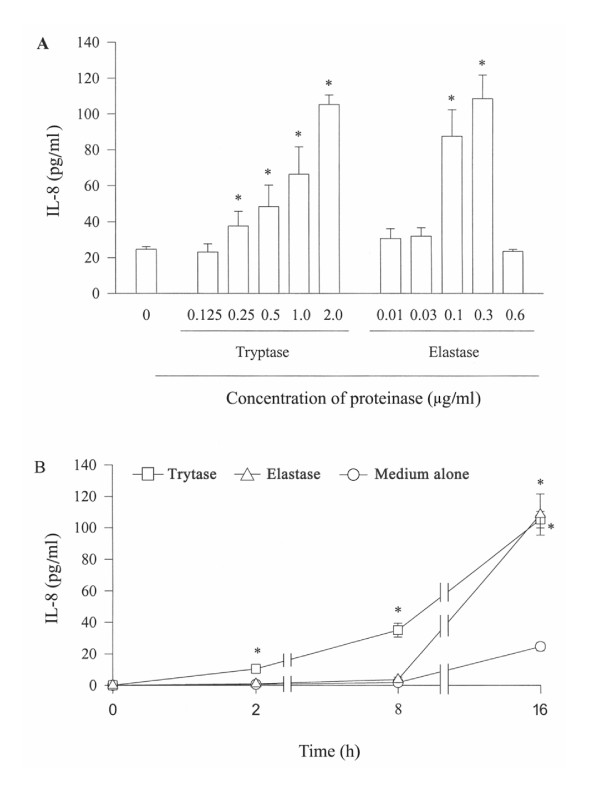
Effect of tryptase and elastase on the release of IL-8 from A549 cells. Cells were incubated with various concentrations of tryptase or elastase at 37°C for 16 h (A), or with 2 μg/ml tryptase or 0.3 μg/ml elastase for 2 h, 8 h and 16 h (B). Values shown are mean ± SE for 5 separate experiments. * *P *< 0.05 compared with the response to medium alone control.

Tryptase at the concentrations from 0.125 to 2 μg/ml induces a concentration dependent IL-8 secretion from A549 cells. Approximately 4.3 fold increase in release of IL-8 is observed when 2 μg/ml of tryptase was incubated with cells for 16 h, and as little as 0.25 μg/ml tryptase is able to provoke a significant release of IL-8 from A549 cell at 16 h following incubation (Figure 3A). Time course study reveals that increased release of IL-8 induced by tryptase begins within 2 h, and lasts at least to 16 h (Figure 3B). Elastase, however, only at the concentrations of 0.1 and 0.3 μg/ml elicits significant release of IL-8 following 16 h incubation period, and the quantity of IL-8 released from A549 cells in response to 0.3 μg/ml elastase is similar to that induced by 2 μg/ml of tryptase (Figure 3A). Time course study shows that elastase induced release of IL-8 occurs after 8 h incubation and maintains at least to 16 h (Figure 3B).

### Inhibition of IL-8 release induced by proteinases by their inhibitors

Hirudin, a specific thrombin inhibitor is able to inhibit thrombin-induced secretion of IL-8 at both 2 and 16 h following incubation. The maximum inhibition of approximately 89% is observed when 10 U/ml of hirudin was added to cells for 16 h (Table 2). Similarly, specific trypsin inhibitors SBTI and α_1_-antitrysin are able to completely abolish trypsin-induced secretion of IL-8 at both 8 and 16 h following incubation (Table 3). It is observed also that MSACK, an inhibitor of elastase completely abrogates elastase induced release of IL-8 (Table 4). In contrast, inhibitors of tryptase, benzamine and leupeptine are only able to inhibit tryptase induced IL-8 secretion by 47.5% and 6.5%, respectively following 16 h incubation (Table 4). Hirudin (Table 2), SBTI, and α_1_-antitrysin (Table 3), benzamidine, leupeptine and MSACK (Table 4) alone at the concentrations tested have little effect on IL-8 secretion from A549 cells.

**Table 1:** Primer sequences for PARs

Primer		Sequence	Size of product (bp)
PAR-1	sense	5'-CAGCTCCTGGCTGACACTCTTTGTC-3'	500
	antisense	5'-CGAGCAGGGTTTCATTGAGCACAT-3'	
PAR-2	sense	5'-GCAGCCTCTCTCTCCTGCAGTGG-3'	461
	antisense	5'-CTGAGGCAGGTCATGAAGAGAATGG-3'	
PAR-3	sense	5'-GGCTGGACAGGAGCCACGAT-3'	403
	antisense	5'-AGCGGTTGATGCTGATGCAGG-3'	
PAR-4	RT	5'-TGAGTAGCTGGGATTACAG-3'	542
	sense	5'-AACCTCTATGGTGCCTACGTGC-3'	
	antisense	5'- CCAAGCCCAGCTAATTTTTG-3'	
β-actin	sense	5'-GTTGCGTTACACCCTTTCTT-3'	148
	antisense	5'-ACCTTCACCGTTCCAGTTT-3'	

**Table 2:** Effect of hirudin on IL-8 release from A549 cells induced by thrombin

Compound (U/ml)	IL-8 released (pg/ml)	
	2 h	16 h
Medium alone	18.7 ± 1.6	64.8 ± 7.2
Thrombin 3	40.1 ± 11.1	181 ± 32
Hirudin 3	18.4 ± 1.7	52.8 ± 5.7
Thrombin 3 + Hirudin 3	21.9 ± 7.2*	73.9 ± 12.9*
Thrombin 10	65.3 ± 18.4	478 ± 131
Hirudin 10	18.9 ± 2.1	59.3 ± 12.6
Thrombin 10 + Hirudin 10	26.8 ± 6.7*	119 ± 16.6*

Data shown are mean ± SE for five separate experiments. Thrombin, hirudin, and thrombin with hirudin were added to cells for 2 h or 16 h. *P < 0.05 compared with the corresponding concentration of thrombin alone.

**Table 3:** Effect of trypsin inhibitors on IL-8 release from A549 cells induced by trypsin

Compound (μg/ml)	IL-8 released (pg/ml)	
	8 h	16 h
Medium alone	173 ± 54	581 ± 91
SBTI 10	154 ± 47	631 ± 102
SBTI 30	194 ± 52	646 ± 100
α_1_-AT 10	nd	505 ± 105
Trypsin 0.1	294 ± 78	993 ± 185
Trypsin 0.1 + SBTI 10	176 ± 47*	567 ± 110*
Trypsin 0.1 + SBTI 30	173 ± 49*	590 ± 120*
Trypsin 0.1 + α_1_-AT 10	nd	556 ± 133*
Trypsin 0.3	718 ± 147	1061 ± 366
Trypsin 0.3 + SBTI 10	158 ± 45*	543 ± 122*
Trypsin 0.3 + SBTI 30	256 ± 67*	654 ± 141*
Trypsin 0.3 + α_1_-AT 10	nd	579 ± 131*

Data shown are mean ± SE for five separate experiments. Trypsin, inhibitor, and trypsin with inhibitor were added to cells for 8 h or 16 h. * *P *< 0.05 compared with the corresponding concentration of trypsin alone. α_1_-AT = α_1_-antitrypsin, SBTI = soybean trypsin inhibitor, nd = not done.

**Table 4:** Effect of the inhibitors of tryptase or elastase on tryptase or elastase induced release of IL-8 from A549 cells

Compound (μg/ml)	IL-8 released (pg/ml)
Medium alone	24.6 ± 1.5
Tryptase 2.0	105.2 ± 5.3*
Elastase 0.3	108.4 ± 13.2*
Benzamidine 30	37.6 ± 4.4
Leupeptine 30	27.4 ± 5.6
MSACK 30	22.1 ± 4.3
Tryptase 2.0 + Benzamidine 30	66.9 ± 4.8^†^
Tryptase 2.0 + Leupeptin 30	100 ± 1.7
Elastase 0.3 + MSACK 30	26.9 ± 3.6^†^

Data shown are mean ± SE for five to six separate experiments. Stimulus, inhibitor, or stimulus with inhibitor was incubated with cells for 16 h. * *P *< 0.05 compared with the response to medium alone; ^†^*P *< 0.05 compared with the corresponding stimulus alone.

### Expression of PARs by A549 cells

**Figure 4:**
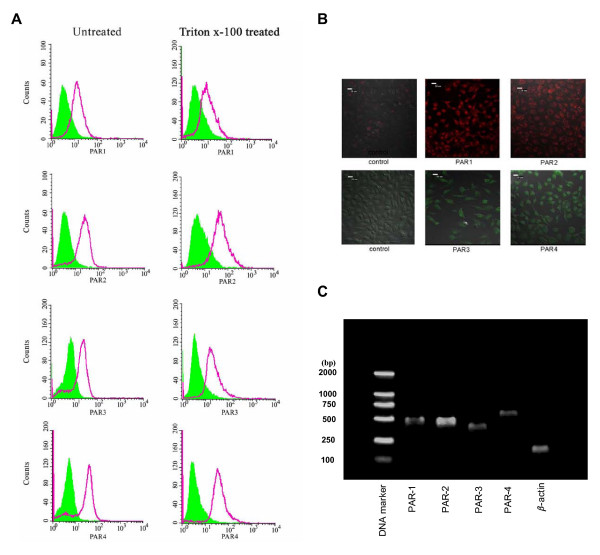
Analysis of expression of PARs on A549 cells by flow cytometry (**A**), Immunofluorescent microscopy (**B**) and RT-PCR (**C**). For PAR-1 and PAR-2 labeling, cells were incubated with PE-conjugated mouse anti-human PAR-1 monoclonal antibody and FITC-conjugated mouse anti-human PAR-2 monoclonal antibody for 30 min on ice. For PAR-3 and PAR-4 labeling, cells were incubated with rabbit anti-human PAR-3 or PAR-4 polyclonal antibodies for 30 min on ice followed by addition of FITC-conjugated goat anti-rabbit polyclonal antibodies. To detect cytoplasmic PARs, A549 cells were permeabilized with 0.2% Triton X-100 for 5 min at room temperature before analysis. Immunofluorescent microscopy was performed with a laser scanning confocal microscope. For RT-PCR analysis of expression of mRNAs of PARs in A549 cells, the gene products of PARs were separated in 1.5% agarose gels, stained with SYBR Green I Nucleic Acid Gel Stain and photographed under UV light. Lane1-6 represented DNA marker, PAR-1 (500 bp), PAR-2 (461 bp), PAR-3 (403 bp), PAR-4 (542 bp) and β-actin (148 bp), respectively.

FACS analysis shows that A549 cells express all four PARs regardless they are permeabilized or not (Figure 4A). Immunofluorescent cell staining shows that PAR-2 seems mainly stained on the membrane surface of A549 cells, whereas PAR-1, PAR-3 and particularly PAR-4 predominately stained in cytoplasm (Figure 4B). An agarose gel electrophoresis revealed that A549 cells express mRNAs for all four PARs (Figure 4C). The amplified RT-PCR products of PAR-1, PAR-2, PAR-3 and PAR-4 mRNAs were sequenced and they all correspond to published sequences of PAR genes (data not shown).

### Induction of IL-8 release by agonists of PARs

**Figure 5:**
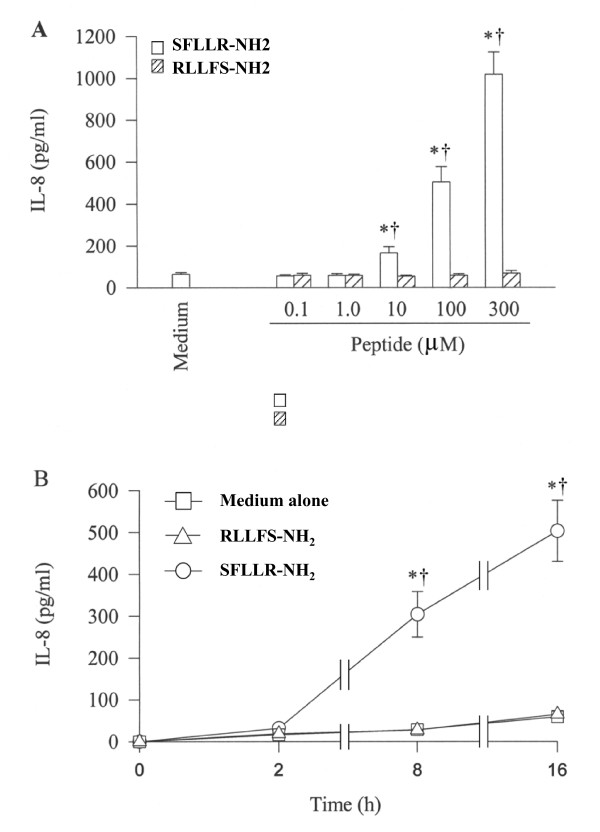
Effect of SFLLR-NH_2_, an agonist peptide of PAR-1 and its reverse peptide RLLFS-NH_2 _on IL-8 release from A549 cells. Cells were incubated (**A**) with various concentrations of SFLLR-NH_2 _(open bar) or RLLFS-NH_2 _(hatched bar) at 37°C for 16 h or (**B**) with 100 μM of SFLLR-NH_2 _and RLLFS-NH_2 _for 2 h, 8 h and 16 h. Values shown are Mean ± SE for five separate experiments performed in duplicate. *P < 0.05 compared with the response to medium alone control; ^†^*P *< 0.05 compared with the response to RLLFS-NH_2 _at the same concentration.

SFLLR-NH_2_, a specific PAR-1 agonist peptide stimulats a concentration dependent secretion of IL-8 from A549 cells following 16 h incubation (Figure 5A), whereas its reverse peptide RLLFS-NH_2 _has no effect on IL-8 release. The maximum release of IL-8 is 15.6 fold induced by 300 μM of SFLLR-NH_2 _following 16 h incubation period (Figure 5A). However, TFRGAP-NH_2_, an agonist peptide of PAR-3 and its reverse peptide PAGRFT-NH_2 _at concentrations 0.1, 1, 10 and 100 μM do not show any influence on IL-8 release from A549 cells following 16 h incubation period (data not shown). The time course study shows that SFLLR-NH_2 _induced release of IL-8 occurs after 8 h incubation and sustains at least until 16 h (Figure 5B).

**Figure 6:**
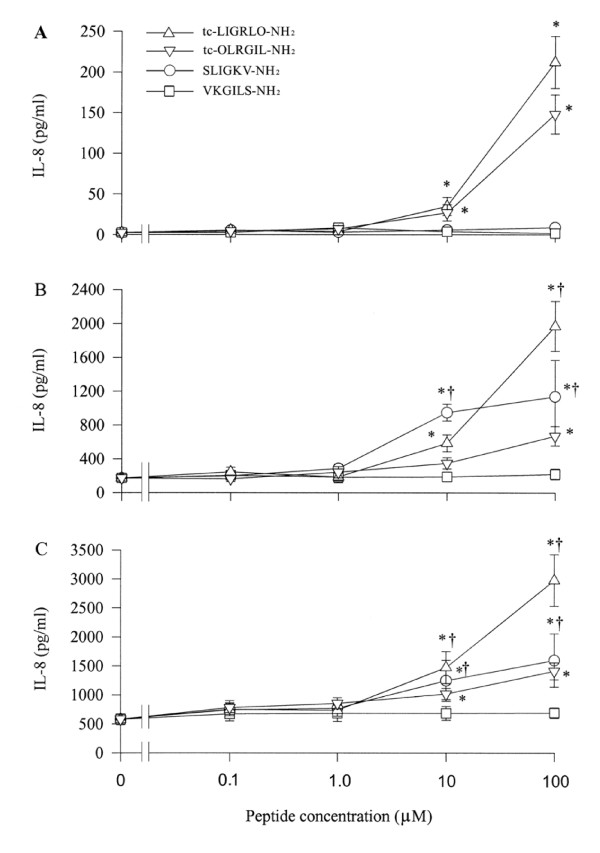
Effect of PAR-2 agonist peptides tc-LIGRLO-NH_2 _and SLIGKV-NH_2 _and their reverse peptides, tc-OLRGIL and VKGILS-NH_2 _on IL-8 release from A549 cells. Cells were incubated with various concentrations of tc-LIGRLO-NH_2 _(open triangle), tc-OLRGIL (open reverse triangle), SLIGKV-NH_2 _(open circle) or VKGILS-NH_2 _(open square) at 37°C for (A) 2 h, (B) 8 h and (C) 16 h. Values shown are Mean ± SE for five separate experiments performed in duplicate. *P < 0.05 compared with the response to medium alone control; ^†^*P *< 0.05 compared with the response to the corresponding reverse peptide at the same concentration.

While SLIGKV-NH_2 _and tc-LIGRLO-NH_2_, two specific agonists of PAR-2 induce concentration dependent secretion of IL-8 from A549 cells following 8 and 16 h incubation periods (Figure 6B,6C) only tc-LIGRLO-NH_2 _is able to stimulate IL-8 release at 2 h (Figure 6A). The maximum release of IL-8 is approximately 79 and 6.6 fold over baseline induced by 100 μM of tc-LIGRLO-NH_2 _at 2 h and 100 μM of SLIGKV-NH_2 _at 8 h, respectively. VKGILS-NH_2 _has little effect on IL-8 release, but tc-OLRGIL-NH_2 _appears to induce a significant release IL-8 from A549 cells. However, the extent of release of IL-8 induced by tc-OLRGIL-NH_2 _is much less than that induced by tc-LIGRLO-NH_2 _(Figure 6). SLIGKV-NH_2 _and tc-LIGRLO-NH_2 _at concentration of 10 μM fail to stimulate IL-10, IL-16, IL-17, and IL-18 secretion from A549 cells following 8 h incubation period (data not shown).

**Figure 7:**
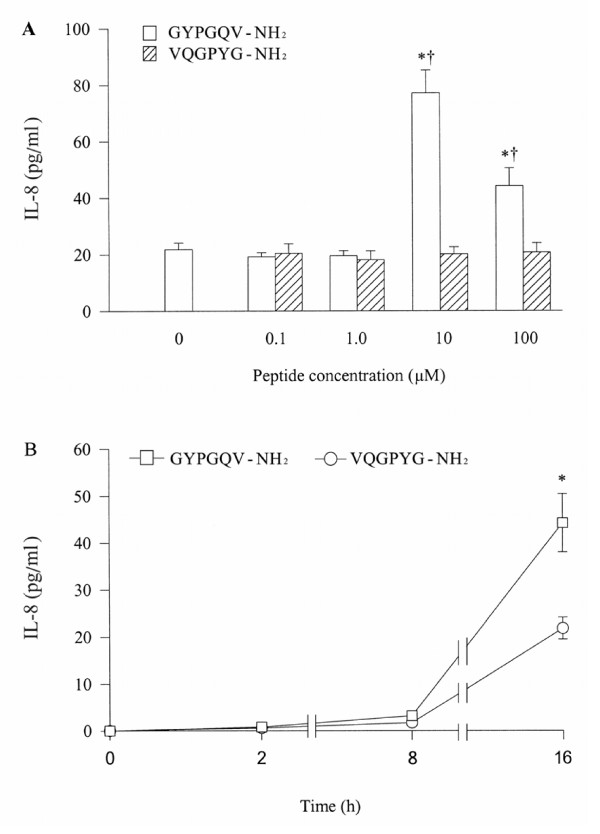
Effect of GYPGQV-NH_2_, a PAR-4 agonist peptide and its reverse peptide VQGPYG-NH_2 _on release of IL-8 from A549 cells. Cells were incubated with various concentrations of GYPGQV-NH_2 _or VQGPYG-NH_2 _at 37°C for 16 h (A), or with 10 μM of GYPGQV-NH_2 _for 2 h, 8 h and 16 h (B). Values shown are mean ± SE for 5 separate experiments. *P < 0.05 compared with the response to medium alone control; ^†^*P *< 0.05 compared with the response to VQGPYG-NH_2 _at the same concentration.

At a concentration of 10 μM, GYPGQV-NH_2_, an agonist peptide of PAR-4 induces a 3.5 fold increase in IL-8 release from A549 cells. However, at a higher concentration (100 μM), it stimulates less IL-8 secretion (Figure 7A). VQGPYG-NH_2_, a reverse peptide of GYPGQV-NH_2_, at the concentrations tested does not show any influence on IL-8 release (Figure 7A). The time course study shows that GYPGQV-NH_2 _induced release of IL-8 occurs after 8 h incubation and sustains at least until 16 h (Figure 7B).

### Effect of serine proteinases and agonists of PARs on expression of IL-8 mRNA in A549 cells

**Figure 8:**
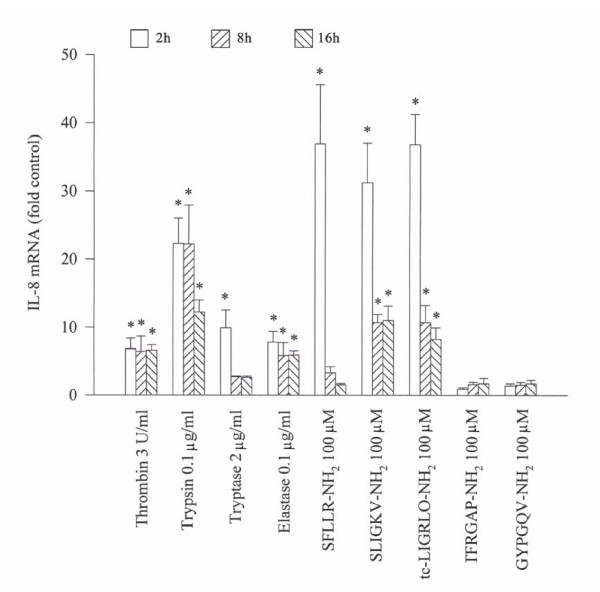
Effect of serine proteinases and agonist peptides of PARs on the expression of IL-8 mRNA in A549 cells. Total cellular RNA was isolated, reverse transcribed to cDNA, and the cDNA was used for real-time PCR. Cells were treated with the testing compounds at 37°C for 2 (open bars), 8 (left hatched bars) and 16 h (right hatched bars), respectively. The data were normalized to the housekeeping gene (β-actin gene) and were expressed as mean ± SE fold of control for three separate experiments performed in duplicate. *p < 0.05 compared with the baseline control.

Thrombin, trypsin, tryptase and elastase stimulate an increase in expression of IL-8 mRNA in A549 cells when they were incubated with the cells. However, the tryptase induced up-regulation of expression of IL-8 mRNA only lasts for 2 h, whereas thrombin, trypsin (declined after 8 h) and elastase provoked expression of IL-8 mRNA continues until 16 h. Up to 6.8, 22.3, 9.9 and 7.8 fold increase in expression of IL-8 mRNA is observed with thrombin, trypsin, tryptase and elastase, respectively following incubation with A549 cells (Figure 8). Dramatically enhanced expression of IL-8 mRNA is found when SFLLR-NH_2_, SLIGKV-NH_2 _or tc-LIGRLO-NH_2 _was incubated with A549 cells for 2 h. At 8 and 16 h following incubation, however, IL-8 mRNA expression induced by SFLLR-NH_2_, SLIGKV-NH_2 _or tc-LIGRLO-NH_2 _is greatly decreased (Figure 8). GYPGQV-NH_2 _and TFRGAP-NH_2 _at 100 μM has little influence on IL-8 mRNA expression in A549 cells (Figure 8). At the concentration of 100 μM, RLLFS-NH_2_, VKGILS-NH_2_, tc-OLRGIL-NH_2_, PAGRFT-NH_2 _and VQGPYG-NH_2_, the reverse peptides of agonists of PARs have little effect on IL-8 mRNA expression in A549 cells (data not shown).

## Discussion

It is demonstrated that human serine proteinases including thrombin, tryptase, elastase and trypsin are potent stimuli of IL-8 secretion from A549 cells, which suggests that they are likely to play a role in IL-8 related airway inflammatory disorders such as asthma, chronic obstructive pulmonary disease and cystic fibrosis.

As little as 5.6 nM of thrombin is able to stimulate approximately 2 fold increase in IL-8 secretion, and 56 nM of thrombin induces 8 fold increase in IL-8 release, indicating that this proteinase is a potent secretagogue of IL-8 release from A549 cells. Human mast cell tryptase, an established mediator of inflammation [18] at a concentration as low as 3.7 nM induces twice more IL-8 secretion over baseline release, and trypsin, a potential mediator of airway inflammation [19] at a concentration of 0.042 nM provokes approximately 3 fold increase in IL-8 secretion from A549 cells, suggesting that tryptic enzyme in airways may play a role in stimulation of IL-8 hypersecretion from airway epithelium. Similarly, elastase, a well-established mediator of airway inflammation at a concentration of 10.2 nM elicits 4.4-fold increase in IL-8 release, indicating that it is a potent secretagogue of IL-8 release from A549 cells as well. At a concentration of 345 nM, elastase was also found to be able to induce IL-8 and MCP-1 secretion from human gingival fibroblasts [9]. However, at the concentrations higher than 62.5 nM elastase could disarm PAR-2 within 10 min following incubation with human lung epithelial cells [20]. These findings suggest that elastase at lower concentrations induce cytokine release from A549 cells, but at higher concentrations may inactivate PAR-2 on human lung epithelial cells including A549 cells. It was impossible for us to examine the effect of elastase at the concentrations higher than 62.5 nM with our experimental system as at the concentration of 0.6 μg/ml (20.4 nM), elastase was able to dissociate A549 cells from plate after 8 h incubation and the suspended cells died soon after (assessed by trypan blue staining). This phenomenon may explain the reason for which elastase at the concentration of 0.6 μg/ml fails to enhance IL-8 release. The similar phenomenon is also observed with trypsin at the concentrations higher than 1 μg/ml. These findings implicate that the detachment of bronchial epithelium observed in chronic airway inflammation may result from the hydrolytic activities of elastase and trypsin. Time course study shows that IL-8 release induced by thrombin, tryptase and trypsin initiates within 2 h following incubation, whereas the response to elastase occurs only after 8 h incubation period. This indicates that elastase and the other proteinases tested may adopt different mechanisms in induction of IL-8 release from A549 cells. The concentrations of tryptase and elastase used in the present study should be achievable under pathological conditions as the level of tryptase in asthmatic bronchial alveolar lavage fluid was 13.2 ng/ml [21] and the levels of elastase in asthmatic and cystic fibrosis sputum were 27 and 466 ng/ml, respectively [22]. While information on the levels of thrombin and trypsin in respiratory fluids are not available, a report described that trypsin-like activity was 46.9 mU/ml in mucoid sputum from patients with asthma [23] might implicate that the concentrations of thrombin and trypsin used in the current study ought to be achievable in the inflammatory airways.

A549 cells have been reported to secrete some 10 pg/ml of elafin and 3 ng/ml of secretory leukocyte protease inhibitor (SLPI) following 24 h incubation [24]. This concentration of elafin, an inhibitor of elastase should not affect the action of elastase on A549 cells, but the concentrations of SLPI, an inhibitor of trypsin and elastase may reduce the stimulatory action of the lower concentrations of trypsin or elastase on A549 cells.

Hirudin inhibites approximately 87% thrombin induced IL-8 secretion; SBTI and α_1_-antitrypsin completely abolish trypsin induced IL-8 secretion and MSACK eliminats 97% elastase induced IL-8 secretion, suggesting strongly that the actions of these proteinases on A549 cells are dependent upon their intact catalytic sites. Since the known substrates of these proteinases on cells are PARs, the expression of PARs on A549 cells was investigated in the present study. To our surprise, benzamidine and leupeptine at a concentration of 30 μg/ml (a quite high concentration for the study on cells based on our previous work [15] are only able to inhibit tryptase induced IL-8 secretion by 47.5% and 6.5%, which suggests that IL-8 secretion induced by tryptase may not depend on its enzymatic activity, and there may be a receptor other than PARs being involved in the process. The similar findings on tryptase were observed previously in other studies [25]. However, to our knowledge, this is the first work examining the effects of thrombin, trypsin, tryptase and elastase on IL-8 release from airway epithelial cells under the same conditions.

It has been reported that a number of human cell types express more than one member of PAR family. Thus, platelets express PAR-1 and PAR-4 genes [14,26], endothelial cells express PAR-1, PAR-2, and possibly PAR-3 [13,27], fibroblast express PAR-1, PAR-2, PAR-3 and PAR-4 genes [28], smooth muscle cell express PAR-1, PAR-2, and PAR-3 genes [29] and respiratory epithelial cells express PAR-1, PAR-2, PAR-3 and PAR-4 genes and possibly proteins [8]. In the present study, we find that A549 cells express all four PARs at both protein and mRNA levels. Since expression of the PARs was observed under both permeabilized and non-permeabilized conditions, it is most likely that all these four PARs are located in both the cytoplasma and the plasma membrane surface of the cells.

The PAR family consists of a group of four G protein-coupled receptors including PAR-1, PAR-2, PAR-3 and PAR-4. They share a unique mechanism of activation involving the proteolytic cleavage of the receptor by serine proteinases to unmask a new N-terminal sequence, the so-called "tethered ligand" that autoactivates the receptor [30]. Thus, thrombin and trypsin cleavage PAR-1 within the amino-tail (LDPR^41^↓S^42^FLLRN, where↓denotes cleavage), trypsin, tryptase and elastase cleavages PAR-2 at the site of SKGR^34^↓S^35^LIGKV, thrombin also cleavages PAR-3 at the site LPIK^38^↓T^39^FRGAP and PAR-4 at PAPR^47^↓G^48^YPGQV [31]. Synthetic receptor-activating peptides corresponding to the new amino termini of the cleaved receptors can also activate PARs [11]. Thus, SFLLR-NH_2 _[11], SLIGKV-NH_2 _[32] and tc-LIGRLO-NH_2 _[33], TFRGAP-NH_2 _[34], GYPGQV-NH_2 _[35] represented agonists of PAR-1, PAR-2, PAR-3 and PAR-4, respectively in the current study. For comparison, their reverse peptides were used as controls.

SFLLR-NH_2_, tc-LIGRLO-NH_2_, SLIGKV-NH_2 _and GYPGQV-NH_2 _stimulates approximately 15.6, 79, 6.6, and 3.5 fold increase in release of IL-8, implicating that there are appropriate mechanisms to carry out IL-8 release process in response to PAR-1, PAR-2 and PAR-4 activation in A549 cells. However, A549 cells do not show any response (in terms of IL-8 release) to PAR-3 activation. Activation of A549 cells to release IL-8 by agonists of PARs indicates that the actions of thrombin, tryptase, elastase and trypsin on A549 cells are most likely carried out through hydrolytic cleavage of N-termini of PARs. The time course shows that the influence of agonists of PAR-1 and PAR-2 on A549 cells initiates within 2 h following incubation, but the action of agonist of PAR-4 on cells appears only after 8 h incubation. These observations suggest that the actions of thrombin on A549 cells are mainly (if not all) carried out through PAR-1, but not PAR-4, whereas the influence of trypsin on cells is most likely through both PAR-1 and PAR-2. It is hard to understand the slower response of cells to elastase and at least partially enzymatic activity independent actions of tryptase on A549 cells. These obviously require further investigation. Using various concentrations of agonist peptides of PARs to stimulate A549 cells may better reflect the actions of these peptides on the cells, which reinforces the previous finding [8].

Up-regulation of expression of IL-8 gene in A549 cells by thrombin, trypsin, tryptase, elastase, PAR-1 and PAR-2 agonist peptides indicates that IL-8 released from A549 cells induced by these stimuli is most likely being newly generated, rather than pre-stored in the cells. The observation that relatively small quantity of IL-8 was released during the first 2 h of incubation in response to the above stimuli also supports our view. While the influence of tryptase and trypsin on IL-8 gene expression does not appear to have been studied previously, the report which found elastase [36,37] and thrombin [38] up-regulated IL-8 gene expression in human epithelial cells may support our current findings. To our knowledge, this is the first work examining IL-8 gene expression in response to several serine proteinases in epithelial cells under the same conditions. The parallel investigation of the actions of serine proteinases on A549 cells may contribute to easier understanding of the role of these proteinases in regulation of IL-8 gene expression. It is difficult to understand the reason why GYPGQV-NH_2 _does not significantly up-regulate IL-8 gene expression, but stimulates IL-8 release from A549 cells at 16 h following incubation. It could be that the significantly increased IL-8 gene expression occurs between 8 and 16 h incubation period, but we did not examine it.

Induction of inflammatory mediator release from airway epithelial cells by agonists of PARs has been demonstrated previously. Thus, agonist of PAR-1 stimulated platelet-derived growth factor secretion from lung epithelial cells [39]; agonists of PAR-2 stimulated IL-8 secretion from 16 HBE cells [40], GM-CSF and eotaxin release from human pulmonary epithelial cells [41] and matrix metalloproteinase-9 release from A549 and primary cultured small airway epithelial cells [42], and agonist of PAR-4 stimulated IL-8 secretion from human respiratory epithelial cells [8].

Our findings further strengthen the view that through activation of PARs, serine proteinases are actively involved in the pathogenesis of airway inflammation.

However, since A549 cells is not a normal airway epithelial cells, it may not fully represent the events happening in normal airway epithelial cells in response to the stimuli above in real life.

## Conclusion

serine proteinases tested are potent stimuli of IL-8 secretion from A549 cells, and the influence of these proteinases on airway epithelial cells are most likely through activation of PARs. Induction of IL-8 secretion by proteinases indicates that they are likely to contribute to the pathogenesis of airway inflammatory disorders.

Development of proteinase inhibitor drugs may be valuable for treatment of these diseases.

## Methods

### Reagents

Human thrombin, trypsin, soybean trypsin inhibitor (SBTI), α_1_-antitrypsin, leupeptin, benzamidine, paraformaldehyde and bovine serum albumin (BSA, fraction V) were all Sigma products. Nonenzymatic cell dissociation solution (CDS) was obtained from Sigma-Aldich (St Louis, MO, USA). Recombinant hirudin, human neutrophil elastase and MeOSuc-Ala-Ala-Pro-Ala-CMK (MSACK) were obtained from Calbiochem (San Diego, CA, USA). Recombinant human Lung βtryptase was from Promega (Madison, WI, USA). Agonist peptides of PARs, as well as their reverse forms were synthesized (CL Bio-Scientific Inc, Xi An, China). The sequences of the active and control peptides, respectively, were: PAR-1, SFLLR-NH_2 _and RLLFS-NH_2_; PAR-2, SLIGKV-NH_2 _and VKGILS-NH_2 _as well as trans-cinnamoyl (tc)-LIGRLO-NH_2 _and tc-OLRGIL-NH_2_; PAR-3, TFRGAP-NH_2 _and PAGRFT-NH_2_; PAR-4, GYPGQV-NH_2 _and VQGPYG-NH_2_. Tissue culture reagents were purchased from GIBCO (Carlsbad, CA, USA) and Sigma Inc. Human IL-8 ELISA kits were purchased from R&D Systems (Minneapolis, MN). TRIzol Reagent was from Invitrogen (Carlsbad, CA, USA). The RNA-PCR kit was from TaKaRa (DaLian, China). SYBR Green I Nucleic Acid Gel Stain was from BMA Inc (USA). PE-conjugated mouse anti-human PAR-1 monoclonal and FITC- conjugated mouse anti-human PAR-2 monoclonal antibodies, anti-PAR-3 and anti-PAR-4 rabbit polyclonal IgGs were purchased from Santa Cruz Biotechnology (Santa Cruz, CA, USA). FITC-conjugated goat anti-rabbit polyclonal antibody was from BD Pharmingen (San Jose, CA, USA). Platinum^® ^SYBR^® ^Green qPCR kit and Superscript^® ^First Strand System were from invitrogen Corp. (Carlsbad, CA, USA). Most of other reagents such as salt and buffer components were analytical grade and obtained from Sigma (St. Louis, MO, USA). The human lung carcinoma cell line A549 (Morphology: epithelial) was obtained from the American Type Culture Collection (Manassas, VA, USA).

### Cell cultures

A549 cells were grown in Dulbecco's modified Eagle's medium (DMEM), supplemented with 10% (v/v) fetal calf serum (FCS), 100 U/ml penicillin and 100 μg/ml streptomycin. Cells were cultured in 75-cm^2 ^tissue culture flasks (Falcon) at 37°C in a 5% (v/v) CO_2_, water-saturated atmosphere.

### Stimulation of epithelial cells

Cells were detached from culture flasks using trypsin, seeded into 12-well tissue culture plates, and grown to about 80% confluence. The cells were then cultured with the serum-free basal medium for an additional 16 h before challenge. For challenge experiments, cells were exposed to various concentrations of thrombin (0.01 – 10 U/ml, 1 U/ml = 5.6 nM), trypsin (1 – 300 ng/ml, 1 μg/ml = 42 nM), tryptase (0.125 – 2 μg/ml, 1 μg/ml = 7.4 nM), elastase (0.01 – 0.6 μg/ml, 1 μg/ml = 34 nM) and their peptidase inhibitors, respectively, and also to PAR-1, PAR-2, PAR-3 and PAR-4 agonist peptides, as well as their reverse peptides (0.1 – 100 μM), respectively. Culture supernatants (1 ml per well) were collected at different times (2 h, 8 h, 16 h), centrifuged at 4°C, and stored at -80°C until use. Concentrations of IL-8 in the A549 culture supernatants were quantified using commercial specific ELISA kits according to the manufacturer's instructions.

### Identification of expression of mRNA of PARs

The expression of mRNA of PARs by A549 cells was investigated with RT-PCR technique. Total RNA was isolated using TRIzol reagent according to the manufacturer's instructions. Briefly, cells were lysed directly by adding 1 ml of TRIzol Reagent to a 3.5 cm diameter dish (1 ml per 10 cm^2^). A total of 200 μl of chloroform was added, and tubes were then centrifuged at 12,000 g for 15 min at 4°C, after which the aqueous phase was transferred to new tubes, RNA was precipitated by adding 0.5 ml of isopropyl alcohol, and then centrifuged at 12,000 g for 10 min at 4°C. Finally, 1 ml of 75% (v/v) ethanol was added to the pelleted RNA, and centrifuged at 7,500 g for 5 min at 4°C. Total RNA was quantified by measuring absorbance ratios at 260/280 nm. cDNA was prepared by reverse transcriptase using a commercial RNA-PCR kit, and reactions were performed according to the manufacturer's instructions. For each reaction, 1 μg of total RNA was reversely transcribed using oligo-d (T) and PAR-4 RT primers according to the protocol. The cDNA was amplified using forward and reverse specific primers for amplifying human PARs. β-actin was used as an internal control. Primers for PAR-1, PAR-2 and PAR-3 were designed based on PAR sequences in Genbank using Omiga software; Primer for PAR-4 was designed as described by Kahn et al [26]. Primers were prepared by Invitrogen Biotechnology Co., Ltd. The primer sequences were summarized in Table 1. The conditions for amplification were: for PAR-1, PAR-2, and PAR-3, the PCR mixture was heated at 94°C for 2 min followed by 35 cycles at 94°C for 30 sec, 67°C for 30 sec, 72°C for 1 min and 72°C for 10 min for 1 cycle; for PAR-4 and β-actin, the PCR mixture was heated at 94°C for 2 min followed by 35 cycles at 94°C for 30 sec, 55°C for 30 sec, 72°C for 30 sec and 72°C for 10 min for 1 cycle. Electrophoresis was conducted in 1.5% agarose gels that were stained with SYBR Green I Nucleic Acid Gel Stain and photographed under UV light. PCR products were then sequenced.

### Quantitative real-time PCR

IL-8 mRNA expression in A549 cells was determined by real-time PCR following the manufacture's protocol. Briefly, total RNA was isolated from the stimulated A549 cells using TRIzol Reagent. cDNA was synthesized from 5 μg of total RNA by using Superscript first strand synthesis system for RT-PCR and oligo-dT primers. A double-stranded DNA binding dye method was used for quantitative PCR/RT-PCR. Real-time PCR was performed in the ABI Prism 7700 Sequence Detection System (Perkin Elmer Applied Systems, Foster City, CA, USA) using the Platinum SYBR Green I PCR kit [each reaction containing 12.5 μl of 2 × SYBR green Master Mix, 300 nM of primers [43,44], 5 μl of a 1:10 dilution of the cDNA or plasmid DNA and water to a total of 25 μl]. The thermal cycling conditions included an initial denaturation step at 95°C for 2 min, 40 cycles at 95°C for 15 s, 60°C for 30 s, and 72°C for 30 s. Consequently, at the end of the PCR cycles, the real-time PCR products were immediately analyzed using a ramping rate of 0.03°C/s from 60 to 95°C to calculate the dissociation curve to verify the correctness of the amplicons. None-template controls for each primer pair were also included on each reaction plate to check for external DNA contamination. Sequence-specific standard curves were generated using 10-fold serial dilutions of plasmid DNA (10^4^~10^9 ^copies), and then the values for the initial concentrations of unknown samples were calculated by using the software (version 1.7) provided with the ABI 7700 system. IL-8 mRNA expression in each sample was finally determined after correction with β-actin expression. Each measurement of a sample was conducted in duplicate.

### Flow cytometry analysis

Adherent A549 cells were detached from culture flasks using the cell dissociation solution. Cells were pelleted by centrifugation, fixed with 2% paraformaldehyde, and resuspended in 1% BSA/PBS. Cells were incubated directly with 1 in 20 dilution of PE-conjugated mouse anti-human PAR-1 monoclonal and FITC-conjugated mouse anti-human PAR-2 monoclonal antibodies for 30 min on ice, followed by two washes with 1% BSA/PBS. For PAR-3 and PAR-4 staining, cells were incubated with 1 in 20 dilution of rabbit polyclonal antibodies for a period of 30 min on ice. After two washes with 1% BSA/PBS, immunofluorescence staining was performed with 1 in 100 dilution FITC-conjugated goat anti-rabbit polyclonal antibodies for 30 min on ice. Finally, cells were resuspended in PBS and labeled cells were analyzed on a FACS Calibur flow cytometer with the use of CellQuest software (BD Biosciences). For staining cytoplasmic PARs, A549 cells were permeabilized with 0.2% Triton X-100 for 5 min at room temperature.

### Immunofluorescence cell staining

A549 cells were seeded onto 8 chamber microscope slides (Nalgene Nunc chamber slides) and allowed them to adhere overnight. The cells were then washed with PBS with 1% BSA followed by fixation in methanol for 20 min on ice. After which the cells were incubated for 5 min with the same wash buffer. Cells were stained directly with 1 in 20 dilution of PE-conjugated mouse anti-human PAR-1 and PAR-2 monoclonal antibodies for 1 h at room temperature and followed by three 1 min washes with 1% BSA/PBS. For PAR-3 and PAR-4 staining, cells were incubated with 1 in 20 dilution of rabbit polyclonal IgG antibodies for a period of 1 h at room temperature and followed by three 1 min washes with 1% BSA/PBS. A FITC-conjugated goat anti-rabbit polyclonal antibody was then added for 1 h at room temperature and followed by a further 1% BSA/PBS wash. Images were obtained on a Nikon EZ-C1 confocal laser scanning microscope (Japan).

### Determination of IL-8, IL-10, IL-16, IL-17 and IL-18

IL-8, IL-10, IL-16, IL-17 and IL-18 release was measured by using ELISA kits according to the manufacturer's instruction.

### Statistics

Data are expressed as mean ± SEM for the indicated number of independently performed duplicated experiments. Statistical significance between means was analyzed by one-way ANOVA or the Student's *t *test utilizing the SPSS11.0 version. *P *< 0.05 was taken as statistically significant.

## Abbreviations

protease-activated receptors (PARs); granulocyte-macrophage colony stimulating factor (GM-CSF); soybean trypsin inhibitor (SBTI); MeOSuc-Ala-Ala-Pro-Ala-CMK (MSACK); trans-cinnamoyl (tc)

## Authors' contributions

HW carried out the most of experimental work and drafted the Material and Method of the manuscript and supplied all analyzed data.

YZ served as a research assistant and participated in the cellular experiments, ELISA assay and RT-PCR experiments.

SH raised the funds, designed and coordinated the study, sorted out technical problems in the study and wrote the major part of the manuscript.

All authors read and approved the final manuscript.
